# Non-random relations in drug use expressed as patterns comprising prescription and over-the-counter drugs in multimorbid elderly patients in primary care: Data of the exploratory analysis of the multicentre, observational cohort study MultiCare

**DOI:** 10.1080/13814788.2021.1933425

**Published:** 2021-06-16

**Authors:** Caroline Krüger, Ingmar Schäfer, Hendrik van den Bussche, Michael Baehr, Horst Bickel, Angela Fuchs, Jochen Gensichen, Wolfgang Maier, Steffi G. Riedel-Heller, Hans-Helmut König, Anne Dahlhaus, Gerhard Schön, Siegfried Weyerer, Birgitt Wiese, Wolfgang von Renteln-Kruse, Claudia Langebrake, Martin Scherer

**Affiliations:** a Hospital Pharmacy, University Medical Centre Hamburg-Eppendorf, Hamburg, Germany; b Department of Primary Medical Care, University Medical Center Hamburg-Eppendorf, Hamburg, Germany; c Department of Psychiatry, Technical University Munich, Munich, Germany; d Institute of General Practice, University Dusseldorf, Dusseldorf, Germany; e Family Medicine, Institute of General Practice, University Hospital Jena, Jena, Germany; f Family Medicine, Institute of General Practice and Family Medicine, University Hospital of Ludwig-Maximilians-University Munich, Munich, Germany; g Department of Psychiatry and Psychotherapy, University Bonn, Bonn, Germany; h Occupational Health and Public Health, University Leipzig, Institute for Social Medicine, Leipzig, Germany; i Department of Health Economics and Health Services Research, University Medical Centre Hamburg-Eppendorf, Hamburg, Germany; j Family Medicine, Institute of General Practice, Goethe University Frankfurt am Main, Frankfurt am Main, Germany; k Department of Medical Biometry and Epidemiology, University Medical Centre Hamburg-Eppendorf, Hamburg, Germany; l Central Institute of Mental Health, Medical Faculty Mannheim/Heidelberg University, Mannheim, Germany; m Institute of General Practice, WG Medical Statistics and IT-Infrastructure, Hannover Medical School, Hannover, Germany; n Scientific Department, Geriatrics Centre, Albertinen-Haus, University of Hamburg, Hamburg, Germany; o Department of Stem Cell Transplantation, University Medical Center Hamburg-Eppendorf, Hamburg, Germany

**Keywords:** Pharmacotherapy, geriatrics, multimorbidity, polypharmacy, primary care

## Abstract

**Background:**

The elderly population deals with multimorbidity (three chronic conditions) and increasinged drug use with age. A comprehensive characterisation of the medication – including prescription and over-the-counter (OTC) drugs – of elderly patients in primary care is still insufficient.

**Objectives:**

This study aims to characterise the medication (prescription and OTC) of multimorbid elderly patients in primary care and living at home by identifying drug patterns to evaluate the relationship between drugs and drug groups and reveal associations with recently published multimorbidity clusters of the same cohort.

**Methods:**

MultiCare was a multicentre, prospective, observational cohort study of 3189 multimorbid patients aged 65 to 85 years in primary care in Germany. Patients and general practitioners were interviewed between 2008 and 2009. Drug patterns were identified using exploratory factor analysis. The relations between the drug patterns with the three multimorbidity clusters were analysed with Spearman-Rank-Correlation.

**Results:**

Patients (59.3% female) used in mean 7.7 drugs; in total 24,535 drugs (23.7% OTC) were detected. Five drug patterns for men (drugs for obstructive pulmonary diseases (D-OPD), drugs for coronary heart diseases and hypertension (D-CHD), drugs for osteoporosis (D-Osteo), drugs for heart failure and drugs for pain) and four drug patterns for women (D-Osteo, D-CHD, D-OPD and drugs for diuretics and gout) were detected. Significant associations between multimorbidity clusters and drug patterns were detectable (D-CHD and CMD: male: *ρ* = 0.376, CI 0.322–0.430; female: *ρ* = 0.301, CI 0.624–0.340).

**Conclusion:**

The drug patterns demonstrate non-random relations in drug use in multimorbid elderly patients and systematic associations between drug patterns and multimorbidity clusters were found in primary care.

KEY MESSAGES

- This study revealed non-random and systematic relations in drug use – comprising prescription and over-the-counter drugs – expressed as drug patterns for multimorbid elderly patients in primary care in Germany.
- There are strong associations between drug patterns and multimorbidity clusters, which enrich the knowledge about the treatment of multimorbid elderly patients in primary care in Germany.

## Introduction

The number and proportion of elderly people are growing worldwide due to demographic change. While, currently in Germany, 22% of the population is 65 years and older, the percentage of this age group is supposed to increase up to 33% in 2060 [1].

Elderly patients have an increased risk for multimorbidity and struggle with related problems like polypharmacy [2,3]. Polypharmacy is defined as the chronic co-prescription or co-application of different drugs at the same time. Common definitions state a number of five or more drugs [4–7]. Moreover, patients with multiple drug use are at risk for potentially inappropriate prescribing due to increased rates of adverse drug events and drug-drug interactions, possibly leading to prescription cascades and decreased health-related quality of life [6,8,9]. Existing clinical practice guidelines for treating chronic conditions are rarely applicable for multimorbid elderly patients because those patients are usually excluded from clinical trials [10]. In addition, multimorbidity and multiple drug use pose a massive challenge for general practitioners and other health care professionals because most clinical practice guidelines do not focus sufficiently on patients with numerous concurrent diseases [11]. Finding associations between drugs and diseases in treating multimorbid elderly patients is a crucial step to improve the health care needs of those patients.

MultiCare – a multicentre, prospective, observational, cohort study conducted in Germany – was set up to monitor disease interactions, progress and consequences of multimorbidity in elderly patients in primary care [12]. General practitioners (GP) were interviewed about their patients’ health status and morbidities. GP’s patients, among others, were interviewed about morbidities, prescription and OTC medication, health and functional status.

Previously, Schäfer et al. carried out an analysis about multimorbidity clusters of multimorbid elderly patients from the MultiCare cohort. Three multimorbidity clusters were detected, characterising different types of elderly multimorbid patients about their morbidities, socio-economic status and gender [13].

Until now, a comprehensive characterisation of the medication within the cohort of multimorbid elderly patients is still insufficient. In former studies, only prescription drugs were included for analysis and a healthier patient collective is presented by excluding patients from nursing homes and patients diagnosed with dementia, which are usually included in most other studies [4,14]. The objectives of the current study are: (I) to identify the relationships of different drugs or drug groups, including OTC drugs and to express these relationships as drug patterns and (II) to study how these drug patterns associate with previously published multimorbidity clusters in the same cohort [13].

## Methods

### Study design

MultiCare was conducted as a multicentre, prospective, observational cohort study of multimorbid patients in general practice. The study protocol is described in detail by Schäfer et al., but in brief, 158 general practices from eight study centres in Germany (Universities of Bonn, Dusseldorf, Frankfurt/Main, Hamburg, Jena, Leipzig, Mannheim and Munich) took part in the study [12]. Patients were included if they had at least three diagnosed chronic diseases and were between 65 and 85 years old. The following eight exclusion criteria were defined according to the study protocol: (I) nursing home patients, (II) blind, (III) deaf, (IV) patients with dementia, (V) life expectancy of fewer than three months, (VI) insufficient ability to read and speak German, (VII) patients who participate in other studies, (VIII) patients poorly known by the physician. Baseline data collection started in July 2008 and three follow-ups were performed. Each recruitment wave took 15 months. For our analysis, the baseline data collected from 2008 up to 2009 was used. GPs provided a list of all patients born between 01.07.1923 and 30.06.1943 from their medical records. Estimating a response rate of 40–50%, 50 patients from each surgery were contacted by study nurses and the trained scientists, and subsequently screened for inclusion and exclusion criteria. In total, 7172 patients out of 50,786 patients were contacted for informed consent and 3317 patients could be included (total response rate: 46.2%). As 128 patients died before baseline interview took place, 3189 patients remained in the cohort. Trained scientists and study nurses conducted standardised interviews at patients’ homes and at the general GP’s surgeries using printed forms. The interviewers performed a brown bag review to collect all information about prescribed and OTC medication used by the patients within the last three months. The GP’s were interviewed to gain information about patients’ morbidity.

### Ethics

The study was conducted in compliance with the Helsinki Declaration. The study protocol was approved by the Ethics Committee of the Medical Association of Hamburg in February 2008 and amended in November 2008 (Approval-No. 2881) and informed consent was obtained from all individual participants included in the study.

### Drug categorisation

Utilised medicinal products were gathered using brown bag reviews, including OTC medicinal products (non-prescription medicinal products, including vitamin supplements and mineral supplements), gaining information about product name, German national drug code, dosage, pharmaceutical form and frequency. A brown bag review is a practice in which patients aid in medication reviews by putting all their medications in a bag and bringing them to their clinician for review [15]. Combined medicinal products were divided into single drugs, and were counted separately. Finally, single drugs (ATC 5th level) were classified using the official German version of the anatomical therapeutic chemical classification system (ATC) version 2016 [16]. To capture all OTC drugs, we coded homoeopathic and herbal traditional medicinal products under the group name herbal and homoeopathic agents.

Categorisation into prescription or OTC drugs was done following guidelines explained in the German regulation for prescribing medicinal products [17].

### Multimorbidity clusters

The comprehensive description of the method on gaining the multimorbidity clusters and detailed results can be found in the publication of Schäfer et al. [13]. In summary, the patients’ morbidity data were recorded from the GPs medical records. The diseases were classified based on the ICD10 code in 46 standardised diagnosis groups. By exploratory factor analysis three multimorbidity clusters were gained: (I) cardiovascular and metabolic disorders (CMD) cluster (hypertension, heart failure, dyslipidaemia, arrhythmia, diabetes mellitus, gout and other), (II) anxiety, depression, somatoform disorders and pain (ADS/P) cluster (chronic back pain, osteoporosis, asthma/chronic obstructive pulmonary disease and other) and (III) neuropsychiatric disorders (NPS) cluster (stroke, depression, heart failure, urinary incontinence and other). It was concluded that there were two different types of multimorbid elderly patients in the MultiCare cohort: Patients with cardiovascular and metabolic disorders that are often male, more aged and with low socio-economic status and patients with mainly ADS and pain-related morbidity and mostly female.

### Statistical analysis

Descriptive analyses of the cohort were performed by calculating the frequencies of age, gender and collected drugs using Excel 2010 (Microsoft Office 2010 and 2016, Redmond, USA). For the following analysis SPSS 23/24 (IBM, Armonk, USA) was used. The Chi-Square-Test was exploited for calculating the gender dependency of OTC drug use. The Mann–Whitney-*U*-Test was used for determining the correlation between gender and number of taken drugs and the Spearman-Rank-Correlation was applied to analyse the effect of age on the number of drugs (significance level *p* < 0.05).

For exploratory factor analysis and scree plots STATA 12.1 (StataCorp, College Station, USA) was used. Drug patterns were detected using exploratory factor analysis because this technique permits the appearance of one variable in more than one factor [18]. For this analysis, the pharmacological subgroup (ATC 3rd level) was applied and the data were divided according to gender. All ATC codes with a prevalence of at least 5% were included to improve the epidemiological interest. A tetrachoric correlation matrix was used and the patterns were rotated oblimin to allow the data to correlate with each other. This technique was used by Schäfer et al. to gain the multimorbidity clusters, where the exact method is described in detail [13,19]. To determine the number of factors, we extracted the factors with the help of scree plots. An ATC code was associated with a drug pattern when the factorloading was at least 0.25. The extracted factors were valued by two pharmacists, two physicians and a psychologist. Patients were assigned to one pattern when they had at least received two of the included ATC codes and could be assigned to multiple clusters.

To correlate the drug patterns and the previously published multimorbidity clusters the Spearman-Rank-Correlation was employed.

## Results

### Characterisation of the elderly, multimorbid patient collective

The MultiCare cohort includes 3189 patients aged between 65 and 85 years, 59.3% of which were women. We found 25,522 drugs from 875 different ATC 5^th^ level codes. After excluding double ATC codes (2.0%) from single patients, 24,535 drugs (96.1%) were related to an ATC code. Patients used in mean 7.7 (± 3.9) drugs, the median was 7 (range 0 to 29) drugs (number of diagnosed chronic diseases: 7.0 ± 2.0 (13)).

Table 1 depicts the distribution of 24,535 drugs regarding the anatomical main groups (ATC 1st level), the top twenty chemical substances (ATC 5^th^ level) of all patients and their proportion according to gender. The most common prescription drugs were simvastatin (34.9%), hydrochlorothiazide (34.7%) and ramipril (21.8%). The most common OTC drugs were acetylsalicylic acid as an antiplatelet agent (35.6%), magnesium (24.0%) and calcium (17.2%). Altogether, 23.7% of drugs were OTC drugs.

**Table 1.** Distribution of 24,535 drugs according to anatomical main group (ATC 1st level) and the top 20 ATC 5th level drugs (sorted according to their ATC 1st level) of 3189 patients of the MultiCare cohort and their proportion within the 1891 female patients in Germany (multiple use possible) (2008–2009).

ATC 1st level	ATC 5th level drugs	Frequency	Proportion per total number of drugs [%]	Proportion per patient [%]	Frequency for female (Proportion per female [%])
Cardiovascular system		9257	37.7		5162 (55.8%)
	Simvastatin	1114		34.9	526 (47.2%)
	Hydrochlorothiazide	1106		34.7	654 (59.1%)
	Ramipril	695		21.8	347 (49.9%)
	Metoprolol	661		20.7	375 (56.7%)
	Bisoprolol	629		19.7	368 (58.5%)
	Amlodipine	466		14.6	258 (55.4%)
	Torasemide	376		11.8	203 (54.0%)
	Enalapril	330		10.3	188 (57.0%)
	Lisinopril	216		6.8	109 (50.5%)
Alimentary tract and metabolism		5006	20.4		3286 (65.6%)
	Magnesium	765		24.0	541 (70.7%)
	Calcium	548		17.2	454 (82.8%)
	Omeprazole	448		14.0	278 (62.1%)
	Metformin	436		13.7	220 (50.5%)
	Cholecalciferol	392		12.3	337 (86.0%)
Nervous system		2507	10.2		1766 (70.4%)
Blood and blood forming organs		1904	7.8		955 (50.2%)
	Acetylsalicylic acid	1134		35.6	573 (50.5%)
	Phenprocoumon	441		13.8	215 (48.8%)
Musculo-skeletal system		1819	7.4		1186 (65.2%)
	Allopurinol	402		12.6	166 (41.3%)
	Diclofenac	390		12.2	268 (68.7%)
	Ibuprofen	335		10.5	244 (72.8%)
Respiratory system		1361	5.5		770 (56.6%)
Systemic hormonal preparations		999	4.1		773 (77.4%)
	Levothyroxine	616		19.3	504 (81.8%)
Genito-urinary system and sex hormones		568	2.3		241 (42.4 %)
Sensory organs		488	2.0		309 (63.3%)
Dermatologicals		190	0.8		123 (64.7%)
Antineoplastic and immunomodulating agents		154	0.6		122 (72.7%)
Various		106	0.4		67 (63.2%)
Antiinfectives		99	0.4		76 (76.8%)
Herbal and homeopathic agents		72	0.3		54 (75.0%)
Antiparasitic products		5	0.02		5 (100.0 %)

### Subgroup analysis: gender and age

Concerning the detected ATC 5th level drugs, there was no statistically significant difference between the prevalence in men and women. However, women were using significantly more drugs than men (7.9 ± 3.9 vs 7.4 ± 3.8 drugs, *p* = 0.002). The OTC drugs’ proportion was highly significant within the female population (26.8% vs. 20.1%, *p* < 0.001).

There was no difference between the classes of detected drugs (ATC 5th level) and increasing age. Patients aged 65 up to 73.91 years old (median) used in mean 7.3 drugs concurrently while patients at the age of 73.91 up to 85 years used in mean 8.1 drugs at the same time. This allows the conclusion that with increasing age people used significantly more drugs (*p* < 0.001, *ρ* = 0.103).

### Composition, frequencies and overlap of drug patterns

In both gender groups, 14 factors with an eigenvalue of 1 or higher were extracted. Applying scree plots, five factors within the male and four factors within the female population were extracted

Tables 2 and 3 show the composition of the different drug patterns with the associated factorloading. Drug patterns were named after diseases that were commonly treated with the included drugs, as follows: (I) drugs for chronic obstructive pulmonary diseases (D-OPD), (II) drugs for coronary diseases and hypertension (D-CDH), (III) drugs for osteoporosis (D-Osteo), (IV) drugs for heart failure (D-HF) and (V) drugs for pain (D-Pain) and the four drug patterns for women: (I) D-Osteo, (II) D-CDH, (III) D-OPD and (IV) diuretic drugs and drugs for gout (D-DG).

**Table 2.** Loading of factors with eigenvalue ≥ 1 and cumulative percent for ATC 3rd level substances of 1298 male patients in Germany (2008–2009).

Male						
		D-OPD^a^	D-CDH^b^	D-Osteo^c^	D-HF^d^	D-Pain^e^
	Eigenvalue	3.60	3.32	2.59	2.52	1.92
	Cumulative percent [%]	7.61	14.99	22.17	29.22	35.36
ATC 3^rd^ level		Factorloading				
A02B	Drugs for peptic ulcer and gastro-oesophageal reflux diseases					0.60
A10A	Insulins and analogues	−0.32		0.31		
A11C	Vitamin A und D, including combinations of the two			0.83		
A12A	Calcium			0.88		
A12C	Other mineral supplements			0.51		−0.34
B01A	Antithrombotic agents		0.85			
C01A	Cardiac glycosides				0.89	
C01D	Vasodilators used in cardiac diseases		0.46			
C03C	High-ceiling diuretics		0.29	0.32	0.47	
C03D	Potassium-sparing agents				0.34	
C07A	Beta blocking agents		0.62			
C08D	Selective calcium channel blockers with direct cardiac effect	0.55				
C10A	Lipid modifying agents		0.71			
M01A	Antiinflammatory and antirheumatic products, non-steroids					0.48
M05B	Drugs affecting bone structure and mineralisation			0.38	−0.26	
N02A	Opioids					0.77
N02B	Other analgesics and antipyretics					0.62
N06D	Anti-dementia drugs				−0.86	
R03A	Adrenergic inhalants	0.95				
R03B	Other drugs for obstructive airway diseases, inhalants	0.95				

^a^Drugs for obstructive pulmonary diseases; ^b^Drug for coronary diseases and hypertension; ^c^Drugs for osteoporosis; ^d^Drugs for heart failure; ^e^Drugs for pain.

The table expresses the eigenvalue for each factor, their cumulative percent (proportion of variance of the drug data, explainable by the patterns) and the factorloading of the ATC 3rd level substances, whereby factorloadings less than 0.25 were omitted. All ATC 3rd level substances loading with a factorloading of 0.25 or more on one factor were included in one of the described drug patterns. Negative factorloadings ≥ 0.25 express a negative association between the drugs and the drug pattern described.

**Table 3.** Loading of factors with eigenvalue ≥ 1 and cumulative percent for ATC 3rd level substances of 1891 female patients in Germany (2008–2009).

Female					
		D-Osteo^a^	D-CDH^b^	D-OPD^c^	D-DG^d^
	Eigenvalue	4.11	3.75	2.35	1.95
	Cumulative percent [%]	9.14	16.92	24.68	30.85
ATC 3rd level		Factorloading			
A10A	Insulins and analogues		0.33		
A10B	Blood glucose lowering drugs, excl. Insulins	−0.26			
A11C	Vitamin A and D, including combinations of the two	0.96			
A12A	Calcium	0.91			
B01A	Antithrombotic agents		0.60		
C01A	Cardiac glycosides		0.83		
C01D	Vasodilators used in cardiac diseases		0.40		
C03A	Low-ceiling diuretics – thiazides				0.61
C03C	High-ceiling diuretics		0.65		
C03D	Potassium-sparing agents		0.27		0.65
M04A	Antigout preparations				0.75
M05B	Drugs affecting bone structure and mineralisation	0.84			
N04B	Dopaminergic agents			−0.43	
R03A	Adrenergic inhalants			0.94	
R03B	Other drugs for obstructive airway diseases, inhalants			0.96	
S01E	Antiglaucoma preparations and miotics				−0.31

^a^Drugs for osteoporosis; ^b^Drug for coronary diseases and hypertension; ^c^Drugs for obstructive pulmonary diseases; ^d^Diuretic drugs and drugs for gout.

The table expresses the eigenvalue for each factor, their cumulative percent (proportion of variance of the drug data, explainable by the patterns) and the factorloading of the ATC 3rd level substances, whereby factor loadings of less than 0.25 were omitted. All ATC 3rd level substances loading with a factorloading of 0.25 or more on one factor were included in one of the described drug patterns. Negative factorloadings ≥ 0.25 express a negative association between the drugs and the drug pattern described.

**Figure 1.**
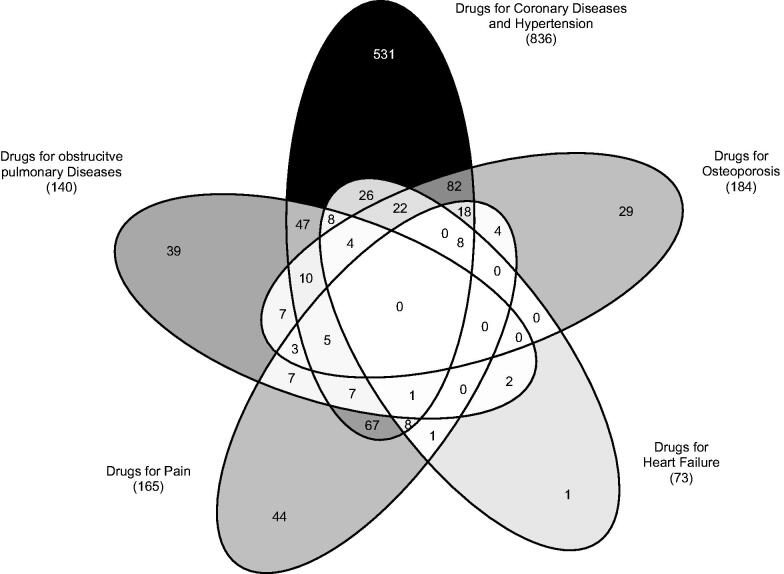
Overlapping of drug patterns (total number of patients) related to the total male population (1298) in Germany (2008–2009).

**Figure 2.**
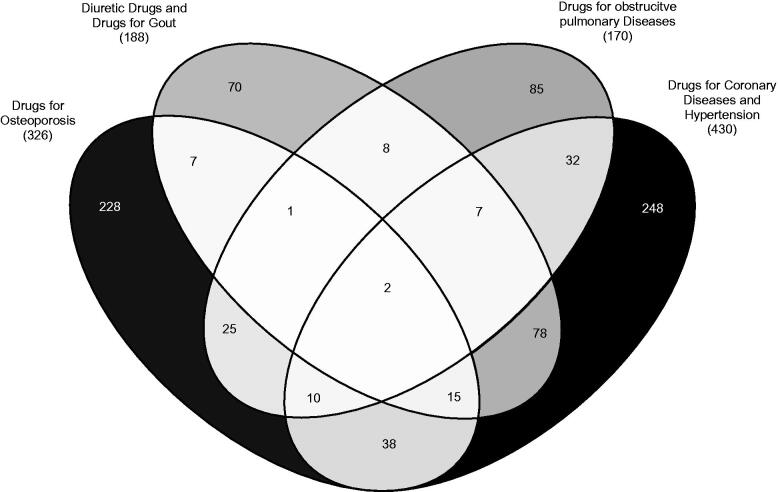
Overlapping of drug patterns (total number of patients) related to the total female population (1891) in Germany (2008–2009).

In total, 75.0% (973) of men and 45.2% (854) of women were relatable to at least one factor. Using this kind of model, a cumulative percent of 35.4% in the male cohort and 30.9% in the female cohort was detectable, expressing the proportion of variance of the drug data that can be explained by the pattern. The overlap of the factors separated by gender is shown in Figures 1 and 2, expressing that 33.8% (329) of men and 26.1% (223) of women could be assigned to at least two patterns. The most prevalent pattern for both genders was the D-CDH pattern (836 [64.4%] for men and 430 [22.7%] for women).

### Comparison of drug patterns and multimorbidity clusters

The correlation between drug patterns and the recently published multimorbidity clusters is shown in Table 4 [13]. There is a moderate and significant correlation between male and female cardiovascular drug pattern and cardiovascular and metabolic disorder multimorbidity cluster (male: *ρ* = 0.376, *p* < 0.001, CI 0.322–0.430, female: *ρ* = 0.301, *p* < 0.001, CI 0.624–0.340). All other detected significant correlations appear with a small effect size (*ρ* < 0.3).

**Table 4.** Correlation coefficients *ρ* according to Spearman, *p*-values and 95% confidence interval of drug patterns and multimorbidity clusters for 3189 patients – separated for male and female – expressing the association between drug patterns and multimorbidity clusters from MultiCare study in Germany (2008–2009).

			95% confidence interval	
	*ρ*	*p*-Value	Min	Max
Male				
D-OPD^a^ x CMD^g^	0.008	0.779	−0.049	0.063
D-OPD x ADS/P^h^	0.088	**0.002**	0.032	0.145
D-OPD x NPS^i^	−0.036	0.191	−0.047	−0.025
D-CDH^b^ x CMD	0.376	**<0.001**	0.322	0.430
D-CDH x ADS/P	−0.089	**0.001**	−0.144	−0.037
D-CDH x NPS	0.031	0.266	−0.021	0.073
D-Osteo^c^ x CMD	0.078	0.005	0.029	0.125
D-Osteo x ADS/P	0.019	0.486	−0.038	0.071
D-Osteo x NPS	0.022	0.434	−0.037	0.087
D-HF^d^ x CMD	0.089	**0.001**	0.052	0.121
D-HF x ADS/P	−0.011	−0.704	−0.061	0.042
D-HF x NPS	0.007	0.804	−0.028	0.068
D-Pain^e^ x CMD	−0.021	0.443	−0.081	0.037
D-Pain x ADS/P	0.103	**<0.001**	0.046	0.161
D-Pain x NPS	0.027	0.325	−0.033	0.101
Female				
D-Osteo x CMD	−0.112	**<0.001**	−0.155	−0.065
D-Osteo x ADS/P	0.102	**<0.001**	0.060	0.142
D-Osteo x NPS	0.002	0.918	−0.041	0.050
D-CDH x CMD	0.301	**<0.001**	0.264	0.340
D-CDH x ADS/P	−0.103	**<0.001**	−0.150	−0.056
D-CDH x NPS	0.232	**<0.001**	0.178	0.284
D-OPD x CMD	−0.003	0.902	−0.048	0.040
D-OPD x ADS/P	0.094	**<0.001**	0.053	0.135
D-OPD x NPS	−0.180	0.436	−0.057	0.026
D-DG^f^ x CMD	0.157	**<0.001**	0.117	0.197
D-DG x ADS/P	0.0001	0.983	−0.045	0.044
D-DG x NPS	0.166	**<0.001**	0.056	0.172

^a^Drugs for obstructive pulmonary diseases; ^b^Drug for coronary diseases and hypertension, ^c^Drugs for osteoporosis; ^d^Drugs for heart failure; ^e^Drugs for pain; ^f^Diuretic drugs and drugs for gout; ^g^Cardiovascular and metabolic disorders; ^h^Anxiety; depression; somatoform disorders and pain; ^i^Neuropsychiatric disorders.

Significant *p*-values are marked in bold.

## Discussion

### Main findings

The 3189 multimorbid elderly patients aged 65 up to 85 years from the MultiCare cohort used 7.7 drugs concurrently. Cardiac drugs and electrolyte preparations like calcium and magnesium are prevalent in the patient cohort. Interestingly women used more OTC drugs than men and a small increased drug use with growing age was detectable.

Our study illustrates characteristic patterns in medication use in multimorbid elderly patients, with a high degree of overlap, pointing out multiple drug use and even polypharmacy. The identified drug patterns for men and women consist of different types of pharmacological subgroups; most of them comprise expectable and non-random drug combinations, exemplified by the D-Osteo and D-CDH pattern.

### Strength and limitations

Even though the data provided by the MultiCare cohort study is from 2008 to 2009, we still have a well selected and representative patient cohort. By taking advantage of the carefully selected inclusion criteria, we can be confident that our results represent the German elderly, multimorbid population. Nonetheless, we might have some regional effects in prescribing because recruitment took place in large cities and rural areas were not covered. However, using the ATC 3rd level drugs is a common procedure to reduce the variability between different study centres [14].

High data quality was provided because interviewers and study nurses were trained and monitored regularly, and standardised interviews were conducted.

A limitation is that we did not know whether all drugs were taken regularly or on-demand. Nonetheless, the brown-bag procedure is a suitable method to collect data about OTC medication use. In this way, we are confident that we did not underestimate the drug use in contrast to most other studies.

The performed factor analysis is an appropriate method with regard to our objectives [20]. Four different methods were used and compared with each other to value the extracted factors and create a significant and reproducible result. We decided to follow the scree plots’ results because they provide the most valid results and are a common method to extract factors [14,18,20,21]. Some relations within the factors could occur because patients were included when they had at least three chronic diseases, which is a higher illness burden than included in most other studies. Nevertheless, in general, 44% of the patients in this age group apply to this definition of multimorbidity.

In contrast to other studies, an additional strength of our study is that patients from nursing homes and patients with dementia were excluded because of their inability to consent, forming a homogenous patient collective. Even though this might impact the generalisability of our data, we are already able to recognise effects like polypharmacy and patterns of drug use in a ‘healthier’ patient collective.

The results presented here are obtained from the baseline assessment of MultiCare cohort study. Longitudinal analysis is needed to confirm the detected drug patterns. Unfortunately, we are unable to explain all associations detected within the drug patterns. Analysis regarding the improvement of the understanding of multimorbidity and drug use in elderly patients is needed.

### Interpretation of study results

Surprisingly, no ACE-inhibitors or angiotensin-II-antagonists were included in any pattern, although most guidelines recommended them for cardiac diseases as first-line therapy. However, as these drugs had a high prevalence (42.5% respectively 22.1%) in the whole cohort, it is possible that they are not specifically loading into one factor. Instead, cardiac glycosides are part of the D-HF pattern. These are recommended as an additional therapy for special indications after incomplete response for chronic heart failure patients [22,23]. Data collection started in July 2008, so it is possible that cardiac glycosides were still more common for the therapy of heart failure than nowadays, although guidelines barely changed since 2008 [24].

D-DG pattern – consisting of diuretic drugs and anti-gout preparations – demonstrates an association between the use of diuretics and gout, and that thiazides and thiazide-like diuretics significantly increase the risk of developing gout [25]. Furthermore, patients with renal insufficiency have a risk for increased uric acid levels.

The D-Pain pattern was only detectable for the male population. As women in our cohort used pain medication with a higher proportion than men, we assume that the frequent use of pain medication in the female population leads to the missing pain dimension.

The high degree of overlap between the drug patterns revealed that even in a presumed healthier patient collective, patients are already at risk for multiple drug use and even polypharmacy and are at risk for the associated negative consequences.

The distinct drug patterns can be associated with the multimorbidity clusters detected by Schäfer et al. [13]. Although we were only able to show this association with a small effect size – except for the higher association between the cardiovascular patterns in both gender groups – they are non-random and enrich the knowledge about the treatment of diseases. The different numbers of included patients might explain the small effect size and the multimorbidity clusters comprise a broader spectrum of diseases than drugs included in the drug patterns.

### Comparison with other studies

Our data (mean: 7.7 drugs per patient) is in good accordance with published data about multimorbid elderly patients, Diez-Manglano et al. found a mean 8.2 (± 3.4) drugs per patient (mean age 81.0 ± 8.8 years) [26]. Studies showing lower numbers of drugs per patient usually did not include OTC drugs [4,14]. A study including OTC drugs found 23.4% OTC drugs per patient, confirming our findings of 23.7% [27]. Although we did not differentiate between dietary supplements and OTC drugs, we could show that women use more OTC drugs than men, which is in good accordance with literature and may result from higher health consciousness in women [28,29]. The slight increase in drug use with increasing age might be attributed to the fact that our cohort does not comprise patients living in nursing homes. As shown, patients residing in nursing homes use more drugs than patients living on their own [30].

Our results regarding the drug patterns are comparable with Calderón-Larrañaga et al., which is the only published study that also employed drug patterns in multimorbid elderly patients [14]. Using the same method as we did (differentiating in using an eigenvalue of 0.3), they detected seven drug patterns, that are quite similar to ours (cardiovascular-, depression-anxiety-, acute respiratory infection-, COPD-, rhinitis-asthma-, pain- and menopause pattern). Interestingly they detected a missing pain dimension in the female population, too.

Another study describing polypharmacy and morbidity patterns excluded patients older than 65 due to multicollinearity. Nevertheless, they also showed connections between multimorbidity and polypharmacy patterns, revealing similar findings for their age groups [31].

In contrast to other studies, the present MultiCare study focussed on the unique patient collective of multimorbid elderly patients by only including patients 65 years and older. In addition, our study is more outright by including OTC medication which forms a large part of patients’ medication.

### Implications for clinical practice

The high number of drug use and especially OTC drug use, detected among multimorbid elderly patients, points out the risk of drug-related problems and consequent negative influence on medication and patient safety in this vulnerable patient group. By discovering non-random associations of drug use we could confirm the results presented in other studies and successfully reproduce known knowledge about the drug therapy safety of multimorbid elderly patients [14,31]. The present study enriches the relations of multimorbidity and drug use in multimorbid elderly patients; this will help to improve the understanding of healthcare needs of multimorbid elderly patients. Further analysis regarding the adequacy of used medication in elderly multimorbid patients is needed.

## Conclusion

This study points out relationships between prescription and OTC drugs in the multimorbid population aged 65 and older. The identified drug patterns – that partly indicate multiple drug use and polypharmacy due to the high degree of overlap – highlight non-random relations in drug use. By showing associations between drug patterns and multimorbidity clusters, we can gain new knowledge on multimorbid elderly patients’ treatment in primary care and clinical routine. Further, the risk of multimorbidity and also polypharmacy is already visible in a presumed healthier patient collective.

With this study, we want to point out a greater awareness for this highly complex cohort and their treatment to improve the drug therapy of multimorbid elderly patients.

## Supplementary Material

STROBE Checklist
